# Transcriptomic analysis of *Sorghum bicolor* responding to combined heat and drought stress

**DOI:** 10.1186/1471-2164-15-456

**Published:** 2014-06-10

**Authors:** Stephanie M Johnson, Fei-Ling Lim, Aliza Finkler, Hillel Fromm, Antoni R Slabas, Marc R Knight

**Affiliations:** Durham Centre for Crop Improvement Technology, School of Biological and Biomedical Sciences, Durham University, South Road, DH1 3LE Durham, UK; Unilever, Colworth Science Park, MK44 1LQ Sharnbrook, Bedford, UK; Department of Molecular Biology and Ecology of Plants, Faculty of Life Sciences, Tel Aviv University, 69978 Tel Aviv, Israel

**Keywords:** Combined stress, Drought, Heat, Microarray, Sorghum, Transcriptomics

## Abstract

**Background:**

Abiotic stresses which include drought and heat are amongst the main limiting factors for plant growth and crop productivity. In the field, these stress types are rarely presented individually and plants are often subjected to a combination of stress types. *Sorghum bicolor* is a cereal crop which is grown in arid and semi-arid regions and is particularly well adapted to the hot and dry conditions in which it originates and is now grown as a crop. In order to better understand the mechanisms underlying combined stress tolerance in this important crop, we have used microarrays to investigate the transcriptional response of *Sorghum* subjected to heat and drought stresses imposed both individually and in combination.

**Results:**

Microarrays consisting of 28585 gene probes identified gene expression changes equating to ~4% and 18% of genes on the chip following drought and heat stresses respectively. In response to combined stress ~20% of probes were differentially expressed. Whilst many of these transcript changes were in common with those changed in response to heat or drought alone, the levels of 2043 specific transcripts (representing 7% of all gene probes) were found to *only* be changed following the combined stress treatment. Ontological analysis of these ‘unique’ transcripts identified a potential role for specific transcription factors including MYB78 and ATAF1, chaperones including unique heat shock proteins (HSPs) and metabolic pathways including polyamine biosynthesis in the *Sorghum* combined stress response.

**Conclusions:**

These results show evidence for both cross-talk and specificity in the *Sorghum* response to combined heat and drought stress. It is clear that some aspects of the combined stress response are unique compared to those of individual stresses. A functional characterization of the genes and pathways identified here could lead to new targets for the enhancement of plant stress tolerance, which will be particularly important in the face of climate change and the increasing prevalence of these abiotic stress types.

**Electronic supplementary material:**

The online version of this article (doi:10.1186/1471-2164-15-456) contains supplementary material, which is available to authorized users.

## Background

Adverse environmental conditions result in substantial losses to agricultural food production worldwide. In particular, abiotic stresses, which include drought, heat and salinity, are amongst the biggest constraints on crop productivity [1, 2]. These types of abiotic stress are, however, rarely presented individually and crops are often subjected to simultaneous adverse conditions, particularly in arid and semi-arid regions of the world [3]. Such combined stress has been shown in Sorghum, wheat and other grass crops to have an even greater detrimental impact on plant productivity than when each stress is imposed individually [4–6]. Land area affected by combined stress is likely to increase given the anticipated climate changes [7]. The co-incidence of heat and drought stress is therefore likely to become an increasingly common scenario in the future.

As a result of their sessile nature, when faced with adverse conditions, plants alter their biochemical and molecular machinery in order to adapt to the change in their environment. Following the perception of the stress, a signal is relayed to the nucleus *via* complex cellular signalling networks involving second messengers such as reactive oxygen intermediates (ROIs) and calcium, calcium-associated proteins and kinase cascades such as mitogen-activated protein (MAP) kinase cascades [8–11]. This leads to the activation of transcriptional pathways which in turn may lead to changes in the flow of metabolites, induction of stress tolerance genes and physiological changes associated with protection from cellular damage [8–11]. Examples of stress tolerance genes include molecular chaperones such as Late Embryogenesis Abundant (LEA) proteins and Heat Shock Proteins (HSPs) which act to protect proteins and membranes [1]. The changes in response to stress at the transcriptomic level must be modulated both rapidly and with specificity to the particular stress encountered and are of key importance for a plant response which is tailored to its environment.

An analysis of changes at the transcript level can be used to identify new signaling proteins and metabolic processes which are important for providing stress tolerance to plants. The transcriptional response to heat or drought stresses imposed on their own has been extensively studied in a number of plant species [12–15]. These studies have identified particular processes required for stress tolerance. Interestingly, it has been found that a combination of drought and heat stress in Arabidopsis and tobacco results in a unique transcriptional response which cannot simply be extrapolated from the effect of each stress imposed individually [16–18]. Plants therefore have novel responses when presented with combined stress.

Sorghum (*Sorghum bicolor L. Moench*.) is a grain crop which is grown in the arid and semi-arid regions of South Africa, Australia, India and America. It is grown primarily as a food source and is the dietary staple for more than 500 million people [19]. Given that Sorghum thrives under conditions of low water availability and high temperatures it is an excellent model for the study of transcriptomic changes induced to enable tolerance to drought and heat stress. A wealth of research has been performed on Sorghum physiology in order to select for agriculturally beneficial traits, however, until recently molecular characterization has been relatively limited. This has been facilitated in recent years by the sequencing of the Sorghum genome [20]. The transcriptomic response of Sorghum to osmotic stress, induced by PEG, has been reported [21, 22] however there are no published reports using *bona fide* drought-treated samples. No transcriptomic analyses of heat responses or combined heat and drought responses in Sorghum have yet been reported.

Given the previously observed unique transcriptional response to combined stress in Arabidopsis and tobacco, we have investigated changes in gene expression which occur following a similar treatment in Sorghum. The aim of this was to identify important processes/responses required for combined stress tolerance in this important crop particularly adapted to hot and arid environments, as it might offer insight not gained from other species. We have used custom-designed microarrays containing 28585 gene probes based on the latest genome annotations at the time of printing. We have identified sets of genes which are differentially expressed in response to each treatment type, as well as demonstrating that there is specificity of gene expression; specific genes being up- and down-regulated only in response to combined (but not individual) stress. Analysis of these genes suggests that specific processes *e.g.* polyamine synthesis might be involved in tolerance to combined heat and drought stress in Sorghum. This study will be useful for not only improving our understanding of basic stress tolerance mechanisms but also in the development of new stress tolerant Sorghum cultivars.

## Results

### Transcriptomic analysis of Sorghum subjected to drought, heat and combined drought and heat stress

To investigate the changes in gene expression which occur in Sorghum subjected to heat and drought stresses either on their own, or in combination, we carried out transcriptomic analyses using DNA microarrays (Agilent Technologies Ltd) containing 28585 unique gene probes. Drought stress was administered to seedlings by withdrawing water from 14 days after sowing (DAS) whilst the remaining (control) plants were well-watered. Heat shock was carried out by subjecting the seedlings to 50°C for 3 hours, compared to a control treatment of 28°C. The heat shock was conducted at the point at which F_v_/F_m_, which gives an indication of photosynthetic efficiency [23, 24], first started to significantly drop in the drought stressed plants, with respect to the well-watered controls. This was at around 3 days following water withdrawal (Additional file 1: Figure S1). In this way we were able to ensure that the drought stressed plants were experiencing *bona fide* stress when the combined treatment was executed.

### Gene expression responses to drought

**Figure 1:**
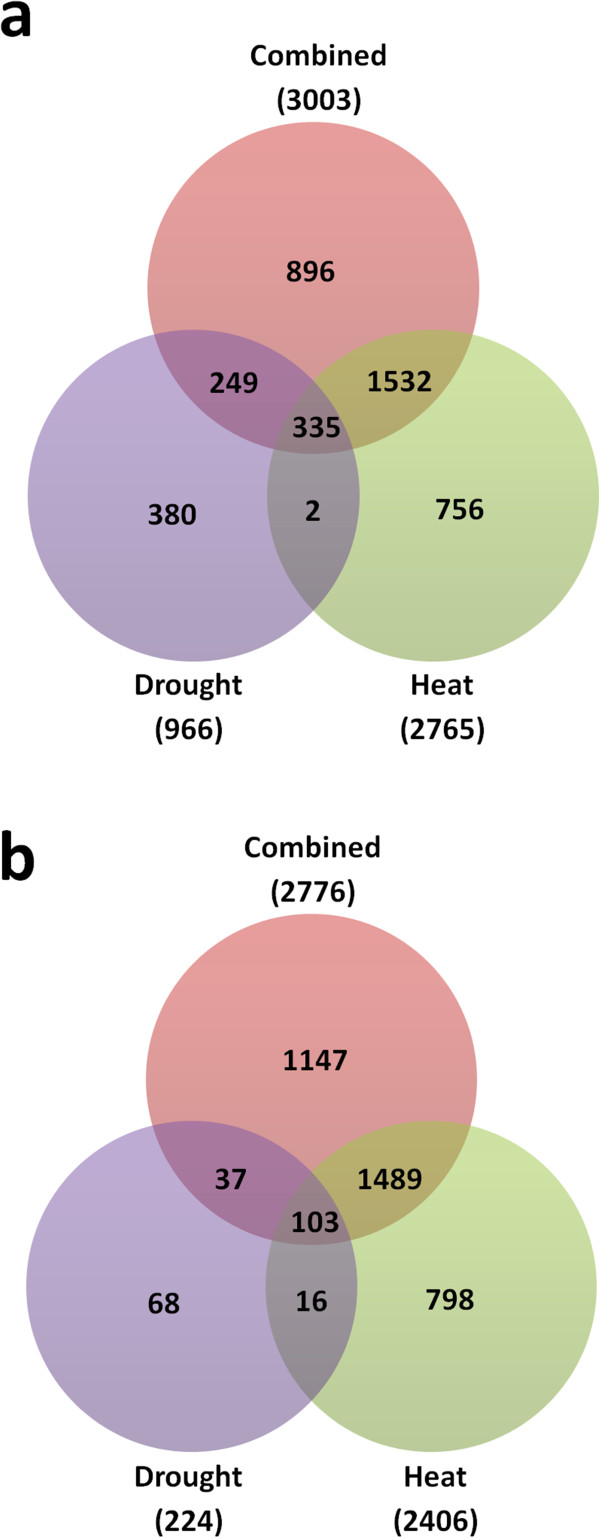
**Venn diagrams showing the number of transcripts up-regulated (a) or down-regulated (b) by either heat, drought or combined heat and drought treatments in Sorghum leaf tissue (compared to control non-stressed plants).** Only transcripts with a change of >2 fold in all 3 replicates were included.

**Figure 2:**
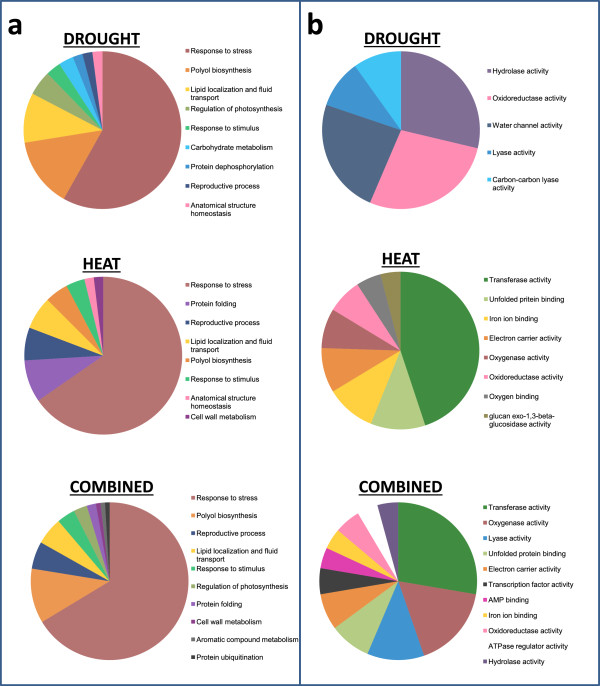
**Pie charts showing summarised Gene Ontology (GO) analysis of the total gene sets responding to either drought, heat or combined drought and heat stress. (a)** shows biological process GO terms and **(b)** shows molecular process GO terms. Only GO terms enriched with a p value of <0.05 were selected and summarized using REVIGO (see methods). Detailed breakdowns of the ontologies are available in Additional file 1: Tables S2, S5 and S8.

**Figure 3:**
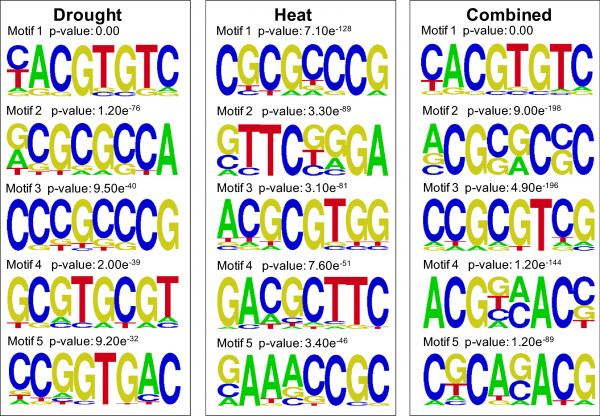
**Most significantly enriched sequences found in promoters of genes up**-**regulated in response to drought (left), heat (centre) and combined heat and drought (right).** Figure shows top 5 statistically-significant consensus sequences generating using AMADEUS and enoLOGOS. Probability values representing significance of enrichment (calculated as described in Methods) are shown for each motif.

As shown in Figure 1 966 Sorghum transcripts were up-regulated and 224 were down-regulated by greater than 2-fold following drought stress only when compared to the untreated plants, equating to approximately 4% of the genes on the chip. Amongst the most highly elevated transcripts are those encoding Late Embryogenesis Abundant (LEA) proteins. Other highly elevated genes include *P5CS2,* which is involved in the metabolism of the compatible solute proline [25] and HKT1, a sodium ion transmembrane transporter involved in maintaining cellular Na^+^ homeostasis [26] (Additional file 1: Table S1). Of the top 100 up and down regulated transcripts however, 15 encode proteins of unknown function (Table 1). In order to further explore the biological processes and molecular functions which are enriched within this (drought-regulated) gene set relative to the background genome, gene ontology (GO) analysis was carried out (Figure 2). In total, 92 GO categories exhibited significant enrichment in the drought up-regulated genes based upon a corrected p-value cut-off of 0.05 (Additional file 1: Table S2). As would be expected, the analysis shows an enrichment of genes involved in response to stress and in particular response to water deprivation. Genes associated with response to ABA are also enriched within the data set which is not surprising given the central role of ABA in the drought stress response [27]. Other examples of enriched GO categories include regulation of photosynthesis, fluid transport and amino acid metabolism (Figure 2a). Promoter motif analysis was carried out to identify promoter motifs which are enriched within the drought data set. As can be seen in Figure 3, the most highly represented promoter motif in the 966 drought up-regulated genes was similar to the abscisic acid response element (ABRE): (C/T)ACGTGTC.

**Table 1:** **Top 100 genes differentially expressed in response to drought (based on average absolute fold change) compared to control unstressed plants**

SbID	Annotation	Average fold change (Abs)	Regulation
Sb01g046000.1	Unknown protein	2900.8	Up
Sb03g029830.1	Unknown protein	1243.2	Up
Sb10g028640.2	Unknown protein	1074.9	Up
Sb07g021850.1	Uknown protein	1066.6	Up
Sb01g046490.1	LEA protein	924.5	Up
Sb09g027110.2	unknown protein	865.4	Up
Sb07g000520.1	CYP71A25	829.8	Up
Sb09g027110.1	LEA protein	776.7	Up
Sb03g001130.1	AAA-type ATPase family protein	669.0	Up
Sb07g023010.1	AMY1 (ALPHA-AMYLASE-LIKE)	586.6	Up
Sb03g011090.1	ATECP63 (EMBRYONIC CELL PROTEIN 63)	570.9	Up
Sb06g004280.1	Transketolase	564.8	Up
Sb02g013190.1	Unknown protein	539.5	Up
Sb02g043300.1	HB-3; transcription factor	520.6	Up
Sb03g034280.1	ATNADP-ME1 (NADP-malic enzyme 1)	467.0	Up
Sb01g012640.1	PAP85; nutrient reservoir	447.1	Up
Sb07g003040.1	Tyrosine decarboxylase	444.5	Up
Sb03g032380.2	Unknown protein	407.1	Up
Sb03g043410.1	Unknown protein	385.5	Up
Sb09g021016.1	AP2 domain-containing transcription factor, putative	383.3	Up
Sb01g009730.1	Unknown protein	367.4	Up
Sb08g023230.1	Unknown protein	367.4	Up
Sb07g003010.1	Tyrosine decarboxylase	349.8	Up
Sb04g031810.1	Unknown protein	348.0	Up
Sb01g037560.1	Mitochondrial import inner membrane translocase subunit Tim17/Tim22/Tim23 family protein	308.4	Up
Sb04g009130.1	LEA domain-containing protein	306.7	Up
Sb01g038670.1	Hydrophobic protein, putative	280.8	Up
Sb01g037560.2	Unknown protein	278.9	Up
Sb03g036980.1	DC1 domain-containing protein	272.5	Up
Sb04g023920.1	UGT85A2 (UDP-glucosyl transferase 85A2)	267.0	Up
Sb09g018420.1	RAB18 (RESPONSIVE TO ABA 18)	264.6	Up
Sb01g050670.1	OLEO1 (OLEOSIN 1)	251.3	Up
Sb08g005220.1	Unknown protein	223.5	Up
Sb10g003700.1	XERO1 (DEHYDRIN XERO 1)	217.0	Up
Sb05g003200.1	Unknown protein	209.3	Up
Sb04g033380.1	HB-7 (HOMEOBOX 7)	192.7	Up
Sb02g006320.1	SIP2 (seed imbibition 2)	190.0	Up
Sb04g021000.1	SAG29 (SENESCENCE-ASSOCIATED PROTEIN 29)	187.4	Up
Sb03g028870.1	KING1 (SNF1-RELATED PROTEIN KINASE REGULATORY SUBUNIT GAMMA 1)	185.3	Up
Sb03g029890.1	PP2CA (PROTEIN PHOSPHATASE 2CA)	184.4	Up
Sb07g026340.1	F-box family protein	179.9	Up
Sb02g034590.1	Aconitate hydratase	177.5	Up
Sb03g030050.1	GBF3 (G-BOX BINDING FACTOR 3)	172.8	Up
Sb06g027900.1	HKT1 (HIGH-AFFINITY K + TRANSPORTER 1)	155.9	Up
Sb10g028640.1	WIN2 (HOPW1-1-INTERACTING 2)	155.3	Up
Sb05g016880.1	unknown protein	147.6	Up
Sb06g001720.1	HAB1 (HOMOLOGY TO ABI1)	144.1	Up
Sb07g015410.1	LEA protein	134.3	Up
Sb06g034080.1	phosphatidylinositol-4-phosphate 5-kinase family protein	131.4	Up
Sb07g021840.1	unknown protein	130.5	Up
Sb03g032230.1	S-adenosyl-L-methionine:carboxyl methyltransferase family protein	129.4	Up
Sb05g005480.1	CYP71B2 (CYTOCHROME P450 71B2)	129.0	Up
Sb04g032890.1	Unknown protein	128.1	Up
Sb04g008300.1	HSFC1	127.0	Up
Sb06g033420.1	Unknown protein	118.5	Up
Sb04g037900.1	DNA-binding family protein	114.9	Up
Sb03g007420.1	Unknown protein	113.3	Up
Sb04g017790.1	LEA protein	105.7	Up
Sb03g012500.1	Mitochondrial import inner membrane translocase subunit Tim17/Tim22/Tim23 family protein	105.6	Up
Sb03g030050.2	Unknown protein	100.8	Up
Sb08g009120.1	Unknown protein	99.0	Up
Sb03g036040.1	HMT2 (HOMOCYSTEINE METHYLTRANSFERASE 2)	93.5	Up
Sb01g017695.1	LTI65 (LOW-TEMPERATURE-INDUCED 65)	91.2	Up
Sb01g036790.1	ECP63 (EMBRYONIC CELL PROTEIN 63)	90.3	Up
Sb06g019610.1	PFK2 (PHOSPHOFRUCTOKINASE 2)	89.6	Up
Sb10g002440.1	Unknown protein	89.2	Up
Sb07g003720.1	TT7 (TRANSPARENT TESTA 7)	89.2	Up
Sb01g039890.1	Protein phosphatase 2C	88.0	Up
Sb04g020543.1	RXF12	86.6	Up
Sb09g023040.1	Phosphatidylethanolamine-binding family protein	86.4	Up
Sb02g004640.1	Unknown protein	85.4	Up
Sb10g000930.1	LEA groUp 1 domain-containing protein	85.2	Up
Sb06g025580.1	unknown protein	84.9	Up
Sb09g006220.1	basic helix-loop-helix (bHLH) family protein	84.6	Up
Sb01g043910.1	HB40 (HOMEOBOX PROTEIN 40)	82.8	Up
Sb02g004560.1	Unknown protein	82.6	Up
Sb06g020045.1	C2 domain-containing protein	80.2	Up
Sb06g027090.1	MLP423 (MLP-LIKE PROTEIN 423)	80.1	Up
Sb03g041320.1	Unknown protein	79.9	Up
Sb09g018630.1	ERF1-2 (EUKARYOTIC RELEASE FACTOR 1–2)	78.6	Up
Sb04g000620.1	BETAFRUCT4; beta-fructofuranosidase	77.7	Up
Sb06g025570.1	Unknown protein	76.2	Up
Sb02g010080.1	AWPM-19-like membrane family protein	74.1	Up
Sb06g025450.1	Unknown protein	72.6	Up
Sb03g032380.1	LEA protein	72.1	Up
Sb09g024255.1	EDL3 (EID1-like 3)	71.2	Up
Sb02g025810.1	Subtilase family protein	70.9	Up
Sb03g006690.1	Unknown protein	70.6	Up
Sb01g030345.1	Plant EC metallothionein-like family 15 protein	70.3	Up
Sb04g016960.1	Unknown protein	69.8	Up
Sb03g039820.2	Unknown protein	69.1	Up
Sb10g008130.1	FTSH6 (FTSH PROTEASE 6)	66.3	Up
Sb01g005110.1	SHY2 (SHORT HYPOCOTYL 2)	66.3	Up
Sb03g039820.1	P5CS2 (DELTA 1-PYRROLINE-5-CARBOXYLATE SYNTHASE 2)	64.4	Up
Sb06g033100.1	Unknown protein	63.9	Up
Sb03g013660.1	Unknown protein	63.1	Up
Sb01g048120.1	Transcription activator-related	60.3	Up
Sb01g020830.1	Peroxidase, putative	59.5	Up
Sb09g020240.1	proton-dependent oligopeptide transport (POT) family protein	59.3	Up
Sb03g035570.1	serine protease inhibitor	58.9	Up

**Figure 4:**
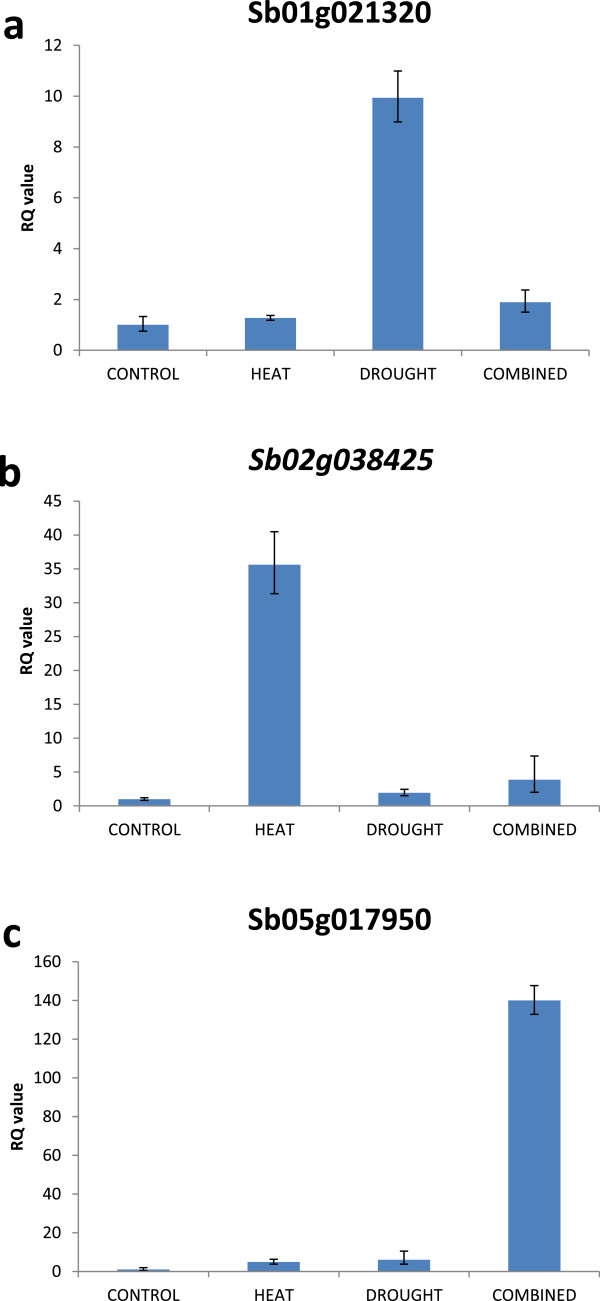
**Relative transcript abundance of genes representative of the gene sets identified as being up-regulated preferentially by either drought stress (a), heat stress (b) or combined heat and drought stress (c).** Error bars represent RQ_MIN_ and RQ_MAX_ and constitute the acceptable error level for a 95% confidence level according to Student’s t-test.

Three hundred and eighty transcripts were found to be up-regulated exclusively in response to drought stress i.e. were not also up-regulated in response to heat, or heat and drought in combination (Figure 1a). This was validated by carrying out qPCR on selected genes and is exemplified by *Sb01g021320* (Figure 4a). These 380 genes include examples associated with lipid transport such as a number of lipid transfer proteins (LTPs) and genes involved with the regulation of cell size such an expansin (see Additional file 1: Table S3 for full gene lists). Interestingly, 2 LEA genes were found to be up-regulated exclusively in response to drought, suggesting specific LEAs may have specific unique roles in response to different stress types.

**Figure 5:**
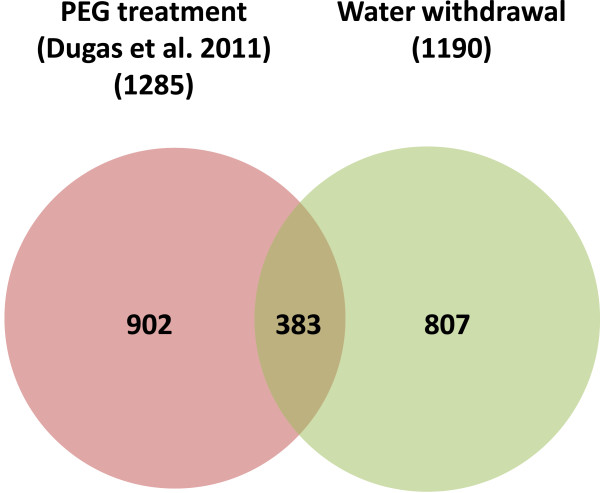
**Venn digaram showing the number of transcripts differentially expressed in response to the gradual drought stress imposed here and in response to the PEG treatment imposed in Dugas et. al **[21]. Only transcripts with a change of > 2 fold are included.

Previous work carried out by Dugas, et al. [21], using next generation sequencing transcriptomic approaches, has identified differentially expressed transcripts in Sorghum leaves following osmotic stress imposed by PEG treatment. In order to determine whether there are differences in the Sorghum response to different types of osmotic stress i.e. PEG treatment compared to the gradual water loss imposed here, we compared the differentially expressed transcripts identified in both studies. Approximately one third of our drought-induced transcripts were in common with those identified by Dugas et al. (Figure 5). GO analysis of these overlapping genes shows an enrichment of genes associated with response to water deprivation, regulation of photosynthesis and response to ABA (Table 2). However, 902 and 807 transcripts were unique to either the PEG treatment or the water withdrawal treatment respectively. GO analysis of the genes unique to the PEG treatment shows an enrichment of genes associated with response to stress and response to reactive oxygen species (Table 3). However, GO analysis of the genes unique to the gradual water withdrawal, shows a strong enrichment of genes associated with wax biosynthesis (Table 4). Different processes therefore seem to be associated with the different stress types.

**Table 2:** **Gene Ontology (GO terms) enriched (p < 0.1) in both the differentially expressed genes following the drought treatment imposed here and also in the PEG treatment imposed in the Dugas et al. **[21]

GO ACCESSION	GO term	P-value	% count in selection	% count in total genome
GO:0009644	Response to high light intensity	0.000	6.23	0.49
GO:0009415	Response to water	0.000	9.51	1.62
GO:0009266	Response to temperature stimulus	0.000	13.77	3.57
GO:0009628	Response to abiotic stimulus	0.000	23.93	9.56
GO:0010205	Photoinhibition	0.000	2.62	0.07
GO:0006950	Response to stress	0.000	28.85	14.70
GO:0006970	Response to osmotic stress	0.000	9.84	2.58
GO:0009409	Response to cold	0.000	9.51	2.45
GO:0042548	Regulation of photosynthesis, light reaction	0.000	2.62	0.12
GO:0042221	Response to chemical stimulus	0.000	25.57	13.05
GO:0009408	Response to heat	0.000	6.56	1.29
GO:0009737	Response to abscisic acid stimulus	0.000	8.85	2.38
GO:0043467	Regulation of generation of precursor metabolites and energy	0.000	2.62	0.21
GO:0042538	Hyperosmotic salinity response	0.002	3.28	0.49
GO:0050896	Response to stimulus	0.002	40.33	28.12
GO:0008287	Protein serine/threonine phosphatase complex	0.004	2.95	0.43
GO:0000302	Response to reactive oxygen species	0.008	3.93	0.86
GO:0009314	Response to radiation	0.010	9.18	3.82
GO:0006470	Protein dephosphorylation	0.018	2.95	0.53
GO:0010119	Regulation of stomatal movement	0.019	2.95	0.54
GO:0023057	Negative regulation of signaling	0.021	2.95	0.55
GO:0004722	Protein serine/threonine phosphatase activity	0.065	3.28	0.79
GO:0009719	Response to endogenous stimulus	0.065	12.79	6.94
GO:0008289	Lipid binding	0.066	3.93	1.12

**Table 3:** **Gene Ontology (GO terms) enriched (p < 0.1) in the differentially expressed genes following the Sorghum PEG treatment carried out in Dugas et al. **[21]** but not in the drought treatment imposed here**

GO ACCESSION	GO term	P-value	% count in selection	% count in total genome
GO:0050896	Response to stimulus	0.000	39.6	28.1
GO:0006950	Response to stress	0.000	23.7	14.7
GO:0009642	Response to light intensity	0.000	3.0	0.7
GO:0010035	Response to inorganic substance	0.000	5.5	2.2
GO:0042221	Response to chemical stimulus	0.000	19.9	13.1
GO:0006805	Xenobiotic metabolic process	0.001	1.2	0.1
GO:0009408	Response to heat	0.001	3.9	1.3
GO:0009410	Response to xenobiotic stimulus	0.002	1.2	0.1
GO:0009607	Response to biotic stimulus	0.002	10.0	5.5
GO:0051707	Response to other organism	0.004	9.3	5.1
GO:0009644	Response to high light intensity	0.005	2.1	0.5
GO:0000302	Response to reactive oxygen species	0.008	2.8	0.9
GO:0005576	Extracellular region	0.008	6.4	3.1
GO:0006026	Aminoglycan catabolic process	0.023	1.1	0.2
GO:0009055	Electron carrier activity	0.023	7.6	4.2
GO:0051704	Multi-organism process	0.027	10.7	6.6
GO:0005385	Zinc ion transmembrane transporter activity	0.029	0.8	0.1
GO:0009628	Response to abiotic stimulus	0.029	14.2	9.6
GO:0071577	Zinc ion transmembrane transport	0.029	0.8	0.1
GO:0006030	Chitin metabolic process	0.032	1.1	0.2
GO:0009719	Response to endogenous stimulus	0.041	10.9	6.9
GO:0061134	Peptidase regulator activity	0.083	1.7	0.4

**Table 4:** **Gene Ontology (GO terms) enriched (p < 0.1) in the differentially expressed genes following the drought treatment imposed here but not in the PEG treatment imposed in the Dugas et al. **[21]

GO ACCESSION	GO term	P-value	% count in selection	% count in total genome
GO:0010025	Wax biosynthetic process	0.065	1.50	0.25
GO:0032787	Monocarboxylic acid metabolic process	0.065	5.26	2.35
GO:0042221	Response to chemical stimulus	0.068	18.80	13.05
GO:0016740	Rransferase activity	0.081	25.26	18.90
GO:0043765	T/G mismatch-specific endonuclease activity	0.081	0.60	0.03
GO:0003824	Catalytic activity	0.084	58.95	51.25
GO:0050896	Response to stimulus	0.084	35.19	28.12

### Gene expression responses to heat

Following heat stress, 2765 Sorghum transcripts were up-regulated and 2406 down-regulated (~18% of the genes on the chip in total) (Figure 1 and Additional file 1: Table S4). The most enriched promoter motif in the 2765 heat up-regulated genes was found to be, CGCGCCCG which, whilst showing some similarity to CAMTA binding sites, was not identical to known promoter motifs. The second and fourth most enriched sequences, however, had consensuses containing the basic 5 bp heat shock element (HSE) motif, nGAAn (nTTCn in reverse complement) and overlapping with the full triple repeat HSE, nGAAnnTTCnnGAAn (Figure 3). As expected, amongst the transcripts most highly up-regulated in response to heat were a large number of genes encoding Heat Shock Proteins (HSPs) (23 of the top 100 gene changes) and Universal Stress Proteins (USPs) (Table 5). Some of these are unique to heat stress with 5 elevated only in response to this stress type (not expressed in response to heat and drought combined, or drought alone). These *HSP* genes are accompanied by the unique up-regulation of 2 heat shock factors (HSFs) which are known to regulate the expression of HSPs (Wang et al., 2003). Genes which are up-regulated only by heat stress are represented by Sb02g038425 (Figure 4b). Other highly induced genes are associated with protection from oxidative stress and include ascorbate peroxidase 3 (see Additional file 1: Table S6 for full gene list). Gene ontology analysis of the heat up-regulated genes shows an enrichment of the following categories: response to heat, response to high light, response to ROI and protein folding (Additional file 1: Table S5). Enriched pathways which are in common with the drought stress response include response to ABA and lipid localization whilst other categories such as protein folding are only enriched in the heat gene set (Figure 2a and Additional file 1: Tables S2 and S5).

**Table 5:** **Top 100 genes differentially expressed in response to heat (based on average absolute fold change) compared to control unstressed plants**

SbID	Annotation	Average fold change (Abs)	Regulation
Sb06g017850.1	HSP22.0	5670.2	Up
Sb03g034390.1	HSP101	4552.7	Up
Sb10g012970.1	Peptidyl-prolyl cis-trans isomerase, putative	1854.5	Up
Sb01g039990.1	HSP18.2	1738.0	Up
Sb01g040000.1	HSP18.2	1567.4	Up
Sb04g034630.1	Universal stress protein (USP) family protein	1510.5	Up
Sb06g016710.1	RAP2.6 (related to AP2 6)	1459.0	Up
Sb09g022400.1	Cytochrome-c oxidase	1437.9	Up
Sb06g000660.1	HSP90.1	1425.2	Up
Sb03g003530.1	HSP17.6II (17.6 KDA CLASS II HEAT SHOCK PROTEIN)	1021.6	Up
Sb01g015760.1	Scarecrow-like transcription factor 9 (SCL9)	1018.0	Up
Sb02g042790.1	Unknown protein	1000.0	Up
Sb03g006920.1	HSP18.2 (heat shock protein 18.2)	984.6	Up
Sb04g007585.1	HSP17.6A (HEAT SHOCK PROTEIN 17.6A)	832.2	Up
Sb04g007600.1	HSP17.6A (HEAT SHOCK PROTEIN 17.6A)	820.9	Up
Sb04g030895.1	Unknown protein	785.0	Up
Sb04g030895.2	Unknown protein	710.6	Up
Sb10g008130.1	FTSH6 (FTSH PROTEASE 6)	676.4	Up
Sb05g021400.1	CYP76C2	512.6	Up
Sb02g026070.1	Unknown protein	491.0	Up
Sb03g006900.1	HSP18.2 (heat shock protein 18.2)	451.6	Up
Sb07g028370.1	HSP21 (HEAT SHOCK PROTEIN 21)	451.2	Up
Sb06g030310.1	Pectinesterase family protein	446.2	Up
Sb08g002950.1	Unknown protein	412.0	Up
Sb01g010460.1	BIP1	364.4	Up
Sb10g007320.1	OPR2	324.5	Up
Sb09g027030.1	Basic helix-loop-helix (bHLH) family protein	318.1	Up
Sb03g006880.1	HSP18.2 (heat shock protein 18.2)	313.4	Up
Sb08g020850.1	Lectin protein kinase, putative	303.8	Up
Sb07g001530.1	Unknown protein	303.7	Up
Sb10g025830.1	Unknown protein	287.7	Up
Sb09g024255.1	EDL3 (EID1-like 3)	261.0	Up
Sb01g015750.1	Unknown protein	253.7	Up
Sb04g030135.1	17.6 kDa class I small heat shock protein (HSP17.6C-CI)	253.0	Up
Sb01g046350.1	HSFA6B	241.9	Up
Sb01g038670.1	Low temperature and salt responsive protein	234.3	Up
Sb01g030345.1	Plant EC metallothionein-like family 15 protein	229.5	Up
Sb02g030040.1	UDP-glucoronosyl/UDP-glucosyl transferase family protein	221.6	Up
Sb05g018030.1	BAG5 (BCL-2-ASSOCIATED ATHANOGENE 5)	213.5	Up
Sb03g006890.1	HSP18.2 (heat shock protein 18.2)	210.9	Up
Sb10g023010.1	MBF1C (MULTIPROTEIN BRIDGING FACTOR 1C)	205.4	Up
Sb05g008770.1	Disease resistance-responsive family protein	204.0	Up
Sb09g023040.1	Phosphatidylethanolamine-binding family protein	203.8	Up
Sb04g027330.1	23.5 kDa mitochondrial small heat shock protein (HSP23.5-M)	197.4	Up
Sb02g026600.1	CYP707A4	195.6	Up
Sb10g007330.1	OPR2	188.9	Up
Sb08g001520.1	No apical meristem (NAM) family protein	188.3	Up
Sb06g001970.1	APX3 (ASCORBATE PEROXIDASE 3)	181.9	Up
Sb03g032910.1	Unknown protein	179.3	Up
Sb03g038160.1	C4H (CINNAMATE-4-HYDROXYLASE)	178.0	Up
Sb03g012940.1	SAG21 (SENESCENCE-ASSOCIATED GENE 21)	177.6	Up
Sb01g027480.1	Unknown protein	170.0	Up
Sb01g008350.1	Glutamate binding	163.9	Up
Sb02g025930.1	GEX1 (GAMETE EXPRESSED PROTEIN 1)	155.6	Up
Sb03g041980.1	Pentatricopeptide (PPR) repeat-containing protein	154.9	Up
Sb09g027890.1	Ferredoxin-related	148.2	Up
Sb10g009970.1	Protein kinase family protein	142.4	Up
Sb10g009090.1	15.7 kDa class I-related small heat shock protein-like (HSP15.7-CI)	140.2	Up
Sb05g027880.1	RCA (RUBISCO ACTIVASE)	137.7	Up
Sb06g016240.1	Nucleic acid binding	134.1	Up
Sb02g025930.2	Unknown protein	129.4	Up
Sb01g040025.1	EDM2; transcription factor	129.4	Up
Sb02g028060.1	Unknown protein	129.0	Up
Sb1058s002010	Unknown protein	119.0	Up
Sb03g005090.1	tRLP7 (Receptor Like Protein 7)	117.8	Up
Sb06g001260.1	ACX4 (ACYL-COA OXIDASE 4)	115.5	Up
Sb01g039436.1	Heat shock cognate 70 kDa protein 2 (HSC70-2)	113.4	Up
Sb09g024180.1	CYC1BAT; cyclin-dependent protein kinase regulator	111.1	Up
Sb07g003040.1	Tyrosine decarboxylase, putative	106.9	Up
Sb07g019840.1	CFIM-25	103.9	Up
Sb01g037590.1	Unknown protein	103.3	Up
Sb01g016900.1	CYP76C1	102.6	Up
Sb07g025210.1	DREB1A (DEHYDRATION RESPONSE ELEMENT B1A)	102.0	Up
Sb02g017220.2	unknown protein	100.3	Up
Sb08g000570.1	unknown protein	98.8	Up
Sb10g003880.1	carboxylesterase/ hydrolase/ hydrolase, acting on ester bonds	98.5	Up
Sb04g024540.1	unknown protein	97.7	Up
Sb10g007430.1	glycine-rich protein	97.6	Up
Sb02g017220.1	Metal ion binding	97.4	Up
Sb03g034980.1	KAT1 (POTASSIUM CHANNEL IN ARABIDOPSIS THALIANA 1)	90.6	Up
Sb01g009370.1	EGY3 (ETHYLENE-DEPENDENT GRAVITROPISM-DEFICIENT AND YELLOW-GREEN-LIKE 3)	90.5	Up
Sb01g039510.1	HSC70-1 (HEAT SHOCK COGNATE PROTEIN 70–1)	90.2	Up
Sb01g037090.1	GolS1 (Arabidopsis thaliana galactinol synthase 1)	89.6	Up
Sb01g014230.1	5PTASE11 (INOSITOL POLYPHOSPHATE 5-PHOSPHATASE 11)	87.2	Up
Sb01g041180.1	HSP21 (HEAT SHOCK PROTEIN 21)	87.1	Up
Sb04g035130.1	17.4 kDa class III heat shock protein (HSP17.4-CIII)	84.7	Up
Sb01g040030.1	HSP17.4	84.5	Up
Sb02g009720.1	Unknown protein	82.8	Up
Sb08g001710.1	MATE efflux family protein	81.6	Up
Sb02g009430.1	UBX domain-containing protein	81.2	Up
Sb01g047480.1	Zinc finger (C3HC4-type RING finger) family protein	79.5	Up
Sb01g039470.1	HSP70 (heat shock protein 70)	77.7	Up
Sb06g027420.1	AOX1B; alternative oxidase	75.1	Up
Sb06g026350.1	Oxidoreductase, 2OG-Fe(II) oxygenase family protein	73.8	Up
Sb10g009140.1	Caleosin-related family protein	73.5	Up
Sb01g003280.1	Zinc finger (C2H2 type) family protein	72.7	Up
Sb06g023160.1	Trypsin and protease inhibitor family protein	72.6	Up
Sb01g004060.1	CYP76C2	72.0	Up
Sb07g016730.1	Unknown protein	70.1	Up
Sb09g030140.1	Glycoside hydrolase family 28 protein	69.4	Up

### Combined heat and drought control the expression of distinct group of genes

Following the combined heat and drought stress 3003 transcripts were up-regulated and 2776 were down-regulated (~20% of gene spots in total) compared to the untreated control. The top 100 gene changes are shown in Table 6 (see Additional file 1: Table S7 for full list). Out of this total of 5779 (both up and down) gene expression changes, a large proportion (60%) were shared with the heat stress only response and 13% were shared with the response to drought (Figure 1). Despite this greater overlap with heat, none of the top 5 promoter motifs enriched in genes upregulated by combined heat and drought contained the basic 5 bp HSE. Indeed the most enriched motif was most similar to the ABRE, (C/T)ACGTGTC (Figure 3). Gene expression changes that were in common in the response to all 3 treatments totalled 438 (335 up-regulated, and 103 down-regulated). These particular genes are associated with the general plant stress response and include heat shock proteins, senescence-associated genes (SAGs) and glutathione transferases (Additional file 1: Table S11). It is not surprising that many of the GO categories enriched following combined stress are in common with those enriched following drought or heat alone (Figure 2). For example, lipid localization and fluid transport, regulation of photosynthesis and protein folding are all enriched in the combined stress gene set (Additional file 1: Table S8). However, some ontological processes appear unique to the combined stress up-regulated transcripts. These include genes associated with protein ubiquitination and aromatic compound metabolism (Figure 2a).

**Table 6:** **Top 100 genes differentially expressed in response to combined heat and drought stress (based on average absolute fold change) compared to control unstressed plants**

SbID	Annotation	Average fold change (Abs)	Regulation
Sb06g017850.1	HSP22.0	17429.6	Up
Sb03g034390.1	HSP101 (HEAT SHOCK PROTEIN 101)	16893.6	Up
Sb10g012970.1	Peptidyl-prolyl cis-trans isomerase	6262.9	Up
Sb04g034630.1	Universal stress protein (USP) family protein	5788.6	Up
Sb04g030135.1	17.6 kDa class I small heat shock protein (HSP17.6C-CI)	5135.6	Up
Sb06g000660.1	HSP90.1 (HEAT SHOCK PROTEIN 90.1)	4744.9	Up
Sb02g042790.1	Unknown protein	4705.1	Up
Sb09g022400.1	Cytochrome-c oxidase	4527.3	Up
Sb01g015760.1	Scarecrow-like transcription factor 9 (SCL9)	3631.6	Up
Sb03g003530.1	HSP17.6II (17.6 KDA CLASS II HEAT SHOCK PROTEIN)	3174.2	Up
Sb03g006920.1	HSP18.2 (heat shock protein 18.2)	3167.3	Up
Sb01g039990.1	HSP18.2 (heat shock protein 18.2)	2929.5	Up
Sb01g010460.1	BIP1	2856.4	Up
Sb05g021400.1	CYP76C2	2799.2	Up
Sb01g040000.1	HSP18.2 (heat shock protein 18.2)	2561.3	Up
Sb06g030310.1	pectinesterase family protein	2467.5	Up
Sb04g007585.1	HSP17.6A (HEAT SHOCK PROTEIN 17.6A)	2375.8	Up
Sb04g007600.1	HSP17.6A (HEAT SHOCK PROTEIN 17.6A)	2356.4	Up
Sb01g046000.1	Unknown protein	2277.8	Up
Sb10g008130.1	FTSH6 (FTSH PROTEASE 6)	2085.7	Up
Sb06g016710.1	RAP2.6 (related to AP2 6)	1584.4	Up
Sb07g028370.1	HSP21 (HEAT SHOCK PROTEIN 21)	1286.9	Up
Sb08g002950.1	Unknown protein	1276.5	Up
Sb03g006900.1	HSP18.2 (heat shock protein 18.2)	1240.0	Up
Sb07g026340.1	F-box family protein	1180.5	Up
Sb05g018030.1	BAG5 (BCL-2-ASSOCIATED ATHANOGENE 5)	1162.8	Up
Sb02g028060.1	Unknown protein	1142.1	Up
Sb08g020850.1	Lectin protein kinase, putative	1128.9	Up
Sb07g021850.1	Unknown protein	1097.8	Up
Sb09g024255.1	EDL3 (EID1-like 3)	994.9	Up
Sb01g038670.1	Hydrophobic protein, putative	981.7	Up
Sb10g028640.2	Unknown protein	976.7	Up
Sb08g000570.1	Unknown protein	924.9	Up
Sb09g027110.2	Unknown protein	815.4	Up
Sb09g027110.1	LEA protein	806.6	Up
Sb09g027890.1	Ferredoxin-related	769.4	Up
Sb03g029830.1	Unknown protein	744.5	Up
Sb10g023010.1	MBF1C (MULTIPROTEIN BRIDGING FACTOR 1C)	700.8	Up
Sb04g032890.1	Unknown protein	694.9	Up
Sb07g001530.1	Unknown protein	664.5	Up
Sb01g015750.1	Unknown protein	661.5	Up
Sb04g030895.1	Unknown protein	636.7	Up
Sb08g023230.1	Unknown protein	625.0	Up
Sb05g008770.1	Disease resistance-responsive family protein	608.9	Up
Sb04g030895.2	Unknown protein	602.7	Up
Sb03g034280.1	NADP-ME1 (NADP-malic enzyme 1)	582.7	Up
Sb09g023040.1	Phosphatidylethanolamine-binding family protein	569.4	Up
Sb03g006880.1	HSP18.2 (heat shock protein 18.2)	567.1	Up
Sb01g030345.1	Plant EC metallothionein-like family 15 protein	536.6	Up
Sb04g035130.1	17.4 kDa class III heat shock protein (HSP17.4-CIII)	515.5	Up
Sb10g009970.1	Protein kinase family protein	506.1	Up
Sb01g039436.1	Heat shock cognate 70 kDa protein 2 (HSC70-2)	489.0	Up
Sb07g000520.1	CYP71A25	452.0	Up
Sb06g004280.1	Transketolase, putative	449.2	Up
Sb02g026070.1	Unknown protein	441.9	Up
Sb06g001970.1	APX3 (ASCORBATE PEROXIDASE 3)	433.7	Up
Sb09g018420.1	RAB18 (RESPONSIVE TO ABA 18)	428.4	Up
Sb04g027330.1	23.5 kDa mitochondrial small heat shock protein (HSP23.5-M)	421.7	Up
Sb09g027030.1	Basic helix-loop-helix (bHLH) family protein	417.0	Up
Sb03g001130.1	AAA-type ATPase family protein	414.0	Up
Sb07g003040.1	Tyrosine decarboxylase, putative	410.9	Up
Sb03g043410.1	Unknown protein	402.9	Up
Sb07g027140.1	Unknown protein	401.4	Up
Sb02g009430.1	UBX domain-containing protein	386.7	Up
Sb01g046490.1	LEA protein	379.2	Up
Sb02g031940.1	FMO1 (FLAVIN-DEPENDENT MONOOXYGENASE 1)	365.8	down
Sb08g001520.1	No apical meristem (NAM) family protein	343.9	Up
Sb03g006890.1	HSP18.2 (heat shock protein 18.2)	343.6	Up
Sb06g023160.1	Trypsin and protease inhibitor family protein	341.9	Up
Sb10g007320.1	OPR2	339.7	Up
Sb01g040025.1	EDM2	337.0	Up
Sb05g003200.1	Phosphatidylethanolamine binding	335.7	Up
Sb07g003010.1	Tyrosine decarboxylase, putative	335.6	Up
Sb01g008350.1	Glutamate binding	332.5	Up
Sb01g026780.1	Unknown protein	322.7	Up
Sb04g031810.1	Unknown protein	320.5	Up
Sb06g016240.1	Nucleic acid binding	318.6	Up
Sb01g046350.1	HSFA6B	310.5	Up
Sb01g009370.1	EGY3 (ETHYLENE-DEPENDENT GRAVITROPISM-DEFICIENT AND YELLOW-GREEN-LIKE 3)	296.4	Up
Sb03g038160.1	C4H (CINNAMATE-4-HYDROXYLASE)	294.8	Up
Sb02g034590.1	Aconitate hydratase	287.0	Up
Sb05g027880.1	RCA (RUBISCO ACTIVASE)	285.7	Up
Sb03g041980.1	Pentatricopeptide (PPR) repeat-containing protein	284.7	Up
Sb08g005220.1	Unknown protein	272.7	Up
Sb01g039470.1	HSP70 (heat shock protein 70)	271.7	Up
Sb07g014620.1	DNAJ heat shock protein, putative	270.1	Up
Sb09g028410.1	DNAJ heat shock family protein	268.3	Up
Sb03g005090.1	AtRLP7 (Receptor Like Protein 7)	260.8	Up
Sb03g012940.1	SAG21 (SENESCENCE-ASSOCIATED GENE 21)	259.2	Up
Sb02g013190.1	Unknown protein	255.0	Up
Sb07g021840.1	Unknown protein	250.7	Up
Sb02g017220.2	Unknown protein	247.7	Up
Sb10g009090.1	15.7 kDa class I-related small heat shock protein-like (HSP15.7-CI)	246.2	Up
Sb02g017220.1	Metal ion binding	243.7	Up
Sb01g034800.1	Nucleic acid binding	242.5	Up
Sb01g042680.1	HSP70T-2 (HEAT-SHOCK PROTEIN 70 T-2)	235.4	Up
Sb02g030040.1	UDP-glucosyl transferase family protein	230.6	Up
Sb10g000930.1	LEA group 1 domain-containing protein	230.2	Up
Sb10g003700.1	XERO1 (DEHYDRIN XERO 1)	230.2	Up
Sb01g000352.1	ERF1 (ETHYLENE RESPONSE FACTOR 1)	224.2	Up

### Identification of genes responding only when drought and heat occur simultaneously

**Figure 6:**
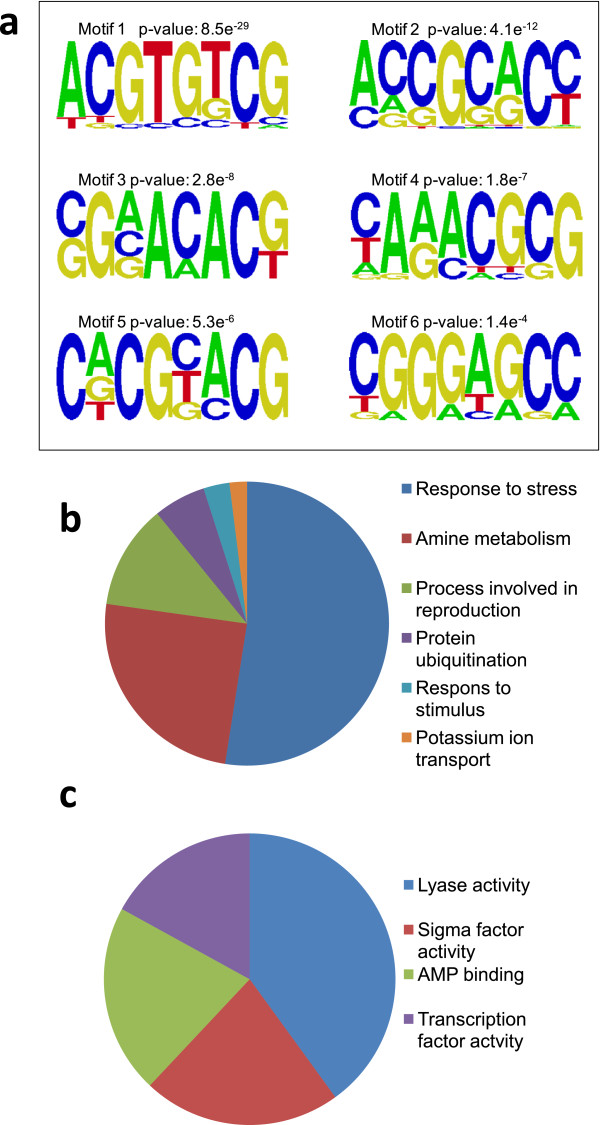
**Analysis of genes up-regulated only by combined heat and drought treatment. (a)** Most significantly enriched sequences found in promoters of genes uniquely upregulated in response to combined heat and drought. Figure shows top 6 statistically significant consensus sequences generating using AMADEUS and enoLOGOS. Probability values representing significance of enrichment (calculated as described in Methods) are shown for each motif. **(b & c)** Ontological analysis showing enriched biological process **(b)** and molecular function **(c)** GO terms p < 0.05. Ontological terms were summarized using the REVIGO tool. Detailed breakdowns of the ontologies are available in Additional file 1: Table S10.

Interestingly, a number of genes, 896 and 1147, were significantly up or down regulated, respectively, *only* in response to combined heat and drought stress (Figure 1). Again, the most enriched promoter motif in the 896 up-regulated genes was an ABRE-like motif, and there was no evidence of HSE-like motifs (Figure 6a). Genes uniquely elevated by combined stress, as exemplified by *Sb05g017950* (Figure 4c) include a number of ion transporters. For example, the potassium transporters *AKT1*, *AKT2/3* and *HAK5* were all (up to 8-fold) and specifically up-regulated. As mentioned earlier there is also specificity in *LEA* and *HSP* expression with, in this case, 2 *HSP* and 3 *LEA* genes being uniquely up-regulated following combined stress. A number of genes encoding signalling proteins and transcription factors were up or down-regulated only by combined stress. These include *ATAF1, MYB78* and *WOX1* amongst others (Additional file 1: Table S9). Genes uniquely down-regulated by combined stress include the transcription factors MYB61 and BZIP61. In addition, there is specificity of calcium-binding proteins with the genes encoding OST1, TCH2, CPK16 and CIPK9 specifically being up-regulated following combined stress. Genes encoding the MAP kinases MKK9 and MPK20 are also uniquely expressed. Ontological analysis of the transcripts uniquely up-regulated by combined stress (Figure 6b and c and Additional file 1: Table S10) showed an enrichment of genes involved in polyamine metabolism and in particular spermidine biosynthesis such as spermidine synthase (*SPDS1*) and S-adenosylmethionine decarboxylase (*SAMDC).* The transcriptomic response of Sorghum to combined heat and drought stress therefore appears unique to that when each stress is imposed individually.

## Discussion

Transcriptomic analyses of plant responses to stress are an effective way in which genes, pathways and processes responsible for plant stress tolerance can be identified. Here, we examined the effect of combined heat and drought stress on the Sorghum transcriptome, using custom designed microarrays containing 28585 individual gene probes. These probes correspond to the latest genome annotation at the time of printing therefore this is the largest microarray study carried out on Sorghum to date.

In response to drought stress we found that expression of ~3.5% of the Sorghum genome was changed by > 2-fold. The genes identified were mostly in known drought-tolerance pathways and there was enrichment of expected promoter motifs. The ABA-responsive element (ABRE) for example, is a known promoter in genes induced by dehydration, salinity and ABA [28]. The number of gene changes identified here is similar to previous studies in which expression of ~4% of the Sorghum genome was changed when subjected to osmotic stress by PEG treatment [21, 22]. Out of our total 1190 drought-induced gene changes 32% were shared with the PEG induced gene changes identified by Dugas et al. using a next generation sequencing transcriptomic approach. There is a significant overlap between the studies although it is clear that the slightly different treatments i.e. a sharp osmotic shock compared to the gradual loss of water have also resulted in the induction of some different response pathways and genes. For example, there is an enrichment of genes associated with response to reactive oxygen species in the transcripts only changed by the PEG treatment whereas there is an enrichment of genes associated with wax biosynthesis in the transcripts only changed by the water withdrawal treatment imposed here. Large quantities of reactive oxygen species (ROI) are generated as an early response to stress [29] therefore reducing ROI levels could be seen as a short term solution to drought. The induction of wax biosynthesis genes on the other hand could result in increased epiculticuar wax which would result in reduced water loss and therefore could be seen as a longer term strategy for survival.

Heat shock resulted in >2-fold changes in expression of 15% of the Sorghum genome. This relatively high level of gene expression changes is not surprising given the acute, severe nature of the heat shock and is comparable to studies in other species [12, 13]. The differentially expressed genes were mostly associated with the heat shock response and again resulted in the expected promoter motifs including CAMTA-like and heat shock elements (HSE) [30].

The combined stress response resulted in 5779 gene changes of which a large proportion were in common with the heat-regulated gene set (60%) and around 13% were shared with the response to drought (Figure 1). Such overlap is understandable: whilst there will be unique challenges presented to the plant when stresses are combined, there is still the need to attend to fundamental issues arising from each stress individually. Reactive oxygen intermediate (ROI) detoxification is required following a number of different stress types [31]. This is displayed here by the enrichment of the GO category ‘response to reactive oxygen species’ in all of the stress types studied (Additional file 1: Tables S2, S5 and S8). Many of the other GO categories enriched by combined stress share elements of those enriched following the other stress types. For example, protein folding is enriched in both the heat and combined stress response and regulation of photosynthesis and water channel activity is also enriched in the drought stress response (Figure 2). It is understandable that similar stresses would require similar downstream processes. This induction of similar pathways can produce cross-tolerance whereby previous exposure to one stress type can provide protection from another [32]. The fact that more genes were found to be in common with the heat stress response is likely to be due the acute nature of the heat shock treatment which results in more synchronised induction of genes.

Despite this large overlap however, there is obvious specificity of gene expression in that there are clear sets of genes which are *only* changed by the combined heat and drought treatment. These specific changes suggest that, similar to what has been found for Arabidopsis and tobacco, Sorghum has a unique transcriptional response to the combined heat and drought stress [16, 17]. Sorghum must therefore be able to perceive this combined stress as a unique environmental condition and reacts to it accordingly. Drought is likely perceived by proteins at the plasma membrane. For example, HK1, a transmembrane histidine kinase is thought to be the first component in relaying an osmotic stress signal to the nucleus [33]. Heat stress is sensed by a complex network of sensors which include plasma membrane proteins and components of the unfolded protein response [34]. The sensing of combined heat and drought would require crosstalk between these sensor systems, or more unlikely, a totally separate sensor for this purpose.

Specificity may also arise at the level of signal transduction. For example, mitogen-activated protein kinase (MAPK) cascades are important signal transducers. These are known to be activated by a number of abiotic stresses and can introduce specificity into a system [35]. Interestingly, there are a number of genes encoding MAP kinases, such as MPK20, which are only elevated by combined stress. Additionally, a number of genes encoding calcium-interacting proteins are specifically elevated such as CPK16. Calcium is an important second messenger and it is thought that unique calcium signatures can result in the expression of unique sets of genes [36]. Calcium binding proteins control these responses and different isoforms have been found to be induced by different plant stresses [10, 37]. It is therefore possible that the CDPK isoforms elevated here are involved specifically in transducing the combined stress signal.

Once in the nucleus unique transcription factors may be responsible for switching on particular sets of genes. A number of transcription factors are only elevated by combined heat and drought (Figure 2). An example transcription factor is ATAF1 which belongs to the NAC family of transcription factors. This has been found to be induced by a number of stresses in Arabidopsis including drought, salinity and wounding [38] therefore it is possible that this has evolved an alternative role in the combined stress response in Sorghum. The HSFC1 transcription factor which is known to induce HSPs in Arabidopsis is also elevated along with some unique HSPs. Other uniquely elevated chaperones include some LEAs which are hydrophilic proteins involved in stress protection. It is possible that these known chaperone molecules contain different motifs which allow recognition and binding of a specific set of molecular targets (Olvera-Carrillo, 2011) and has similarly been shown to be the case in the combined stress response of Arabidopsis [17]. It must be borne in mind however, that these experiments were carried out at one specific time point therefore a detailed time course is required to be able to draw more detailed conclusions.

Amongst other downstream genes regulated by combined stress were those encoding enzymes which are involved in the biosynthesis of polyamines and in particular spermidine such as SAMDC and SPDS1. Polyamines have been implicated in tolerance to multiple stresses including high and low temperature, oxidative stress and salinity [39, 40]. They have been suggested to play a role in ROI scavenging and membrane protection [41]. Perhaps the combined heat and drought treatment results in a higher levels of ROI production than heat and drought treatment individually and therefore higher polyamine levels are required to counteract this. Interestingly, one of the uniquely elevated transcription factors, WOX1, has been shown to physically interact with SAMDC suggesting a possible way in which the activity of this enzyme is regulated by combined stress [42].

## Conclusions

It is clear that there is a degree of plasticity in the Sorghum response to abiotic stress, with evidence for both cross-talk and specificity. This is similar to previous findings in Arabidopsis and tobacco suggesting conservation of mechanisms across species. There are however some elements of the combined stress response which appear unique to Sorghum such as a potential role for polyamine biosynthesis and specific transcription factors and signalling molecules. A functional characterization of these identified genes and pathways is required as they could be used as possible targets for the enhancement of stress tolerance either by marker assisted selection or transgenics. Given the predicted increase in prevalence of drought and heat stress on agricultural land there is a further need to analyse the effect of combined stress on crop species.

## Methods

### Plant growth conditions and stress treatments

Seeds of Sorghum (*Sorghum bicolor L. Moench*.) R16 variety were imbibed overnight in water and surface-sown singly onto soaked 42 mm Jiffy peat pellets (LBS horticulture Ltd, Lancashire, UK). Seedlings were grown in a controlled growth chamber at 28°C day, 23°C night, 12 h photoperiod and set to 0% humidity. Plants were subjected either to control (no treatment), heat, drought or combined heat and drought conditions (6 plants per treatment). These stress assays were developed specifically for Sorghum and are detailed below. Drought stress was applied to the “drought” and “heat and drought” plants by withholding water from 14 days after sowing. At this stage the seedlings had 3 leaves. The remaining plants were well watered. The first visual symptoms of drought stress appeared in the form of leaf curling and slight wilting at 4 days following water withdrawal. This is similar to previous studies in which plants grown under similar conditions showed signs of water stress including a reduction in CO_2_ assimilation and reduced transpiration rate after 4 days of withholding water [43]. Measurements of the ratio of variable fluorescence (F_v_) to maximal fluorescence (F_m_) of plant photosystems can be used as a proxy for the stress status of plants because a reduction in Fv/Fm indicates photoinibition and therefore that a plant is actually experiencing stress [23, 24]. Therefore in order to quantify when drought stress was first starting to have a physiological effect, the F_v_/F_m_ of all of the plants was measured daily using a FluorCam 700mf (Photon Systems instruments, Brno, Czech Republic) on the F_o_, F_m_ and Kautsky effect setting. All plants were dark acclimated for 30 mins prior to measurements. At the timepoint at which the F_v_/F_m_ of the un-watered plants was first significantly lower (error bars showing standard error are no longer overlapping) than that of the watered plants, they were subject to either heat shock by incubation in the dark (to ensure equal levels of light) at 50°C for 3 h (heat and combined treatment) or 28°C for 3 h (control and drought treatment). The youngest 3 leaves were sampled and tissue was pooled for each treatment set. Experiments were carried out in triplicate to give 3 biological replicates. All treatments were carried out at the same time of day for each biological replicate to reduce variation due to circadian/diurnal factors. Tissues samples were harvested into liquid nitrogen and stored at −80°C.

### Microarray design

Custom expression microarrays (4X44K format) for Sorghum were designed and submitted for manufacturing using the Agilent Technologies eArray web-based application (https://earray.chem.agilent.com/earray/). Briefly, Sbicolor release 79 coding sequences were downloaded from http://www.plantgdb.org/SbGDB/, based upon these 29289 CDS sequences, 28585 microarray probes (60 mer oligonucleotides) were designed. In addition, for 10 of the longest CDS, 10 tiling probes were also designed. These probes were randomly laid out onto the 4X44K microarray design format by eArray, along with default Agilent control probes (Agilent Technologies UK Ltd., Wokingham, Berkshire, UK), and 10 additional replicate probes of 100 randomly selected Sorghum CDSs.

### cRNA synthesis and labelling

All products were obtained from Agilent Technologies UK Ltd. (Wokingham, Berkshire, UK) and used according to manufacturer’s protocol unless stated otherwise. Total RNA was isolated using the RNeasy Mini Kit (Qiagen Sussex, UK). The integrity of the RNA was confirmed with analysis by the Agilent 2100 bioanalyzer (Palo Alto, CA) and the Agilent RNA 6000 Nano Kit (Cat no # 5067–1511). RNA (1 μg) was added to 1.2 μL of T7 promoter primer and 5 μL of a “spike-in” control and made to a total volume of 11.5 μL with nuclease free water. The primer and template was denatured at 65°C for 10 mins. The One-Color Low RNA Input Linear Amplification Kit PLUS was used for the synthesis of cRNA as follows: 5 × First Strand Buffer, DTT (to 10 mM), dNTP mix (to 0.5 mM), Moloney murine leukemia virus (MMLV) reverse transcriptase (1 μL stock to 20 μL reaction) and RNaseOut (0.5 μL of stock to 20 μL reaction) were added to the denatured template. The cRNA was synthesized by incubation at 40°C for 2 h and then denaturation at 65°C for 15 minutes. Transcription Buffer (×4), DTT (to 7.5 mM), NTP mix (8 μL stock to 80 μL reaction), PEG (to 4%), RNaseOUT (0.5 μL to 80 μL), inorganic pyrophosphate (0.6 μL to 80 μL reaction), T7 RNA Polymerase (0.8 μL to 80 μL reaction) and Cyanine 3-CTP (2.4 μL to 80 μL reaction) were added. The synthesis of the cRNA was performed by incubation at 40°C for 2 h. The labelled cRNA was purified using the RNeasy Mini Kit (Qiagen, Sussex, UK) according to the manufacturer’s protocol and quantified using a UV–VIS Spectrophotometer.

### Hybridization and washing of microarray slides

The Agilent Hybridization Kit (catalog no. 5188–5242) was used: 2 μg of the labelled sample RNA was added to 10 x blocking Agent, 25 × fragmentation buffer and nuclease free water to total volume of 55 μL. The RNA was fragmented by incubation at 60°C for 30 min. Fragmentation was stopped by the addition of 55 μL 2 × GE Hybridization Buffer HI-RPM. The hybridization was performed for 17 h at 65°C and 10 rpm. Slides were them washed for 1 min in Wash Solution 1; 1 min in Wash Solution 2 (prewarmed to 37°C); and 20 s in acetonitrile. Slides were incubated for 30 s in Agilent Stabilization and Drying Solution (catalog no. 5185–5979). The slides were scanned with the Agilent G2505C Microarray Scanner System (61 × 21.6 mm scan region, 5 μm single pass scanning mode, green dye channel). The accession number for the data series is GSE48205 (data is embargoed for 12 months, but can be accessed from: http://www.ncbi.nlm.nih.gov/geo/query/acc.cgi?token=rxmbxmaiogewefw&acc=GSE48205).

### Bioinformatic analysis

The Agilent Feature Extraction Software (v10.7) was used to extract data from scanned microarray images. The extracted data was analysed using GeneSpring GX 11 (Agilent Technologies, CA, USA). Agilent standard scenario normalizations for FE1-color arrays were applied to the data set. Controls, spots of poor quality and gene probes which were not present in all 3 reps in either the control or treatment samples were excluded from the analysis. This yielded approximately 21000 probes for each control vs. treatment comparison. From these selected genes those with a fold-change of >2 in all 3 reps of each treatment were selected. Gene Ontology term enrichment was determined using agriGO (http://bioinfo.cau.edu.cn/agriGO/) and redundant GO terms were removed using REVIGO (http://revigo.irb.hr/) (medium similarity). Promoter motif analysis was performed using the AMADEUS program (http://acgt.cs.tau.ac.il/amadeus/). Parameters selected for AMADEUS included: promoter length - 1000 bp upstream to the transcription start site; motif length- 8 bp; motif reference database- TRANSFAC. A boot-strapping procedure was performed, which re-runs the entire algorithm on randomly selected gene sets, each with the same size as the real target set, and the lowest *p*-value from each run was recorded. A normal distribution was then fitted to these *p*-values and used to correct the *p*-values of the motifs discovered in the real target set. The corrected score is an estimate of the empirical probability that a motif with the same *p*-value (or lower) could be found in randomly selected gene sets. Fifty cycles of randomizations to obtain fixed p-values were used. The matrices obtained by AMADEUS for each motif were further processed by enoLOGOS (http://biodev.hgen.pitt.edu/cgi-bin/enologos/enologos.cgi), a web-based tool that generates sequence logos.

### Realtime PCR

Quantitative Real Time PCR was used to validate the gene expression data obtained by the microarrays as described previously [44]. cDNA was synthesised from Sorghum RNA using a high capacity cDNA reverse transcription kit (Applied Biosystems, California, USA). qPCR was carried out using an AB 7300 real time PCR system (Applied Biosystems California USA) and Go Taq qPCR master mix (Promega, Wisconsin,USA). Primers were designed using Primer3 (http://frodo.wi.mit.edu/) and synthesized by Invitrogen. Sequences used: Sb03g032380.1 Forward: 5′-TCGGTACTGCTGCAAACAAG-3′ and Sb03g032380.1 Reverse: 3′-CCGTGTTCATCACCTTCTCC-5′; Sb01g021320.1 Forward: 5′-GCGCGTCCGCTATATAATGT-3′ and Sb01g021320.1 Reverse: 3′-CTTGCTGCTGTTGCTGTCTC5′; Sb02g038425.1 Forward: 5′-TGAGGAAGCTTGGGGTAATG-3′ and Sb02g038425.1 Reverse: 3′-CCCATAAGGACGCCAAAGTA-5′; Sb02g003260.1 Forward: 5′-GATGGCTCGATTTCCTTGTC-3′ and Sb02g003260.1 Reverse: 3′-GCCGATGATCTCCTTCTTCA-5′; Sb05g008020.1 Forward: 5′-AAGCGAGCAGTAAACCGTGT-3′ and Sb05g008020.1 Reverse: 3′- GTGATGAGAGGAGGGGAACA-5′; Sb05g017950.1 Forward: 5′- GGCAGCACTAGCAACAACAA-3′ and Sb05g017950.1 Reverse: 3′-GGAAAGTAGCTTCCCCTTGG-5′. These genes were chosen as exemplar genes based on the fact that the microarray data showed that they are highly up-regulated (>2 fold in all 3 reps) following only one of the treatment types i.e. heat, drought or combined stress. Sb03g038910.1 was identified from the microarray as unchanging following each treatment and was therefore used as an endogenous control with the following oligos: Sb03g038910.1 Forward: 5′-AGGTCCTGCTCCAGATCCTC-3′ and Rev: 3′-AAAGGAGAGGGTAGCGGAAG-5′.

### Availability of supporting data

The data set(s) supporting the results of this article are included in the article and are available in the GEO repository, (http://www.ncbi.nlm.nih.gov/geo/query/acc.cgi?token=rxmbxmaiogewefw&ac;GSE48205).

## Electronic supplementary material

Additional file 1: Figure S1: The Fv/Fm (variable fluorescence/maximal fluorescence of Photosystem II) measured daily following water withdrawal. **Table S1** - Genes differentially expressed in response to drought. **Table S2** - Gene Ontology (GO) terms enriched (p < 0.05) in the genes differentially expressed in response to drought. **Table S3** - Genes differentially expressed in response to drought only. **Table S4** - Genes differentially expressed in response to heat. **Table S5** - Gene Ontology (GO) terms enriched (p < 0.05) in the genes differentially expressed in response to heat. **Table S6** - Genes differentially expressed in response to heat only. **Table S7** - Genes differentially expressed in response to combined heat and drought. **Table S8** - Gene Ontology (GO) terms enriched (p < 0.05) in the genes differentially expressed in response to combined heat and drought. **Table S9** - Genes differentially expressed in response to combined heat and drought only. **Table S10** - Gene Ontology (GO) terms enriched (p < 0.05) in the genes differentially expressed in response to combined heat and drought only. **Table S11** - Genes differentially expressed in response to all 3 stress types. (XLSX 1 MB)
